# Medicare and Medicaid enrollment and outside hospitalizations among HIV-infected and uninfected veterans engaged in VA care: a retrospective cohort study

**DOI:** 10.1186/s12913-015-0684-8

**Published:** 2015-01-22

**Authors:** Harry Chang, Janet Tate, Amy C Justice, Michael E Ohl

**Affiliations:** Yale School of Medicine, Yale University, 333 Cedar Street, New Haven, CT 06510 USA; Veterans Aging Cohort Study, VA Connecticut Healthcare System, 950 Campbell Avenue, West Haven, CT 06516 USA; Department of Internal Medicine, Yale School of Medicine, 330 Cedar St, Boardman 110, P.O. Box 208056, New Haven, CT 06520 USA; Department of Public Health, Yale School of Public Health, 60 College Street, P.O. Box 208034, New Haven, CT 06520 USA; VA Office of Rural Health (ORH), Midwest Rural Health Resource Center, Iowa City VAMC, 601 Highway 6 West (152), Iowa City, IA 52246 USA; Center for Comprehensive Access and Delivery Research and Evaluation (CADRE) at the Iowa City VA Medical Center, Mailstop 152, Iowa City VAMC, Iowa City, IA 52246 USA; Department of Internal Medicine, University of Iowa Carver College of Medicine, 200 Hawkins Drive, Iowa City, IA 52242 USA

**Keywords:** HIV, AIDS, Veterans, Medicare, Medicaid, Fragmentation

## Abstract

**Background:**

Many veterans engaged in care with the Veterans Administration (VA) health system are also enrolled in Medicare and/or Medicaid and may receive care both inside and outside of the VA. Use of dual health systems has been associated with worse outcomes. Veterans with HIV may have different rates of Medicare and Medicaid enrollment and may be at greater risk of poor outcomes related to non-VA use. This study compares the frequency and factors associated with Medicare and/or Medicaid enrollment and non-VA use in an HIV-infected and uninfected population of veterans.

**Methods:**

We used data from the VA and Center for Medicare & Medicaid Services from 2004 and 2005 to determine the frequency of Medicare and/or Medicaid enrollment among a cohort of HIV-infected and uninfected veterans engaged in VA care. We then restricted the cohort to veterans enrolled in fee-for-service (FFS) Medicare and/or Medicaid with at least one hospitalization and identified characteristics associated with non-VA hospital admissions.

**Results:**

HIV-infected veterans had higher rates of Medicare and/or Medicaid enrollment than uninfected veterans (38% vs. 33%, p < 0.01), though the opposite was true when our sample was limited to veterans 65 years and older (53% vs. 70%, p < 0.0 1). Among veterans enrolled in the VA and FFS Medicare and/or Medicaid, veterans with HIV had greater illness severity and more frequent hospitalizations, but were less likely to be hospitalized outside the VA (48% vs. 54%, p < 0.01). HIV infection was associated with lower odds of outside hospitalization (OR = 0.76 [95% CI: 0.68, 0.85]).

**Conclusions:**

Veterans with HIV have higher rates of Medicare and/or Medicaid enrollment, but lower odds of non-VA hospitalization. The VA integrated model of HIV care may discourage outside use among HIV-infected veterans.

## Background

A significant proportion of veterans enrolled with the Veterans Administration (VA) healthcare system are also enrolled in Medicare and/or Medicaid (“CMS enrollment”). An expansion of Medicaid coverage under the Patient Protection and Affordable Care Act of 2010 (ACA) will likely increase the proportion of CMS enrolled veterans [1]. Use of non-VA health care (“CMS use”) among veterans already engaged in VA care can lead to fragmented, inefficient, lower quality care and increased mortality [2-6].

Previous work has described CMS enrollment and identified factors that influence CMS use among VA enrolled veterans, particularly veterans 65 years and older that have age qualified for Medicare [7-9]. Less is known about CMS use among veterans under 65 years of age, who may qualify for Medicare through disability, or those enrolled in Medicaid. People living with HIV are more likely to qualify for Medicare through disability than age, and Medicare currently provides coverage for approximately one-fifth of the people receiving HIV care in the U.S. [10]. Medicaid coverage for HIV varies on a state-to-state basis, but it currently covers about 50% of the people receiving HIV care in the U.S. [11]. Little is known about CMS enrollment among HIV-infected veterans, and the frequency and drivers of CMS use among veterans with both VA and CMS coverage.

HIV-infected veterans are an aging cohort vulnerable to adverse outcomes because of the complexity of their care needs, including multimorbidity, polypharmacy, mental health issues, and substance use [12-14]. The VA is the largest provider of care for persons with HIV in the United States, with over 20,000 veterans in care, and has invested significant resources to create an integrated HIV care model [15,16]. The integrated model for HIV care in VA emphasizes case management and access to coordinated medical and behavioral health care services, including mental health care, urgent care, substance abuse and on-site pharmacy services. Previous research has demonstrated that integrated HIV care at the VA can lead to higher rates of viral load suppression [14]. Given the high, and potentially expanding, rates of CMS enrollment in the HIV population, an understanding of Medicare and Medicaid enrollment and the frequency and drivers of CMS use among veterans with HIV is necessary to inform efforts to coordinate care.

The objectives of this paper were to: 1) determine the frequency of Medicare and/or Medicaid enrollment among HIV-infected veterans engaged in VA care, compared with uninfected veterans; 2) determine the frequency of non-VA hospital admissions among male, CMS enrolled HIV-infected and uninfected veterans with any hospitalizations; and 3) identify characteristics that predict non-VA hospital admission among male HIV-infected veterans enrolled in Medicare and/or Medicaid, compared with uninfected veterans. We hypothesized that CMS enrolled HIV-infected veterans would be less likely to use non-VA care than their uninfected counterparts.

## Methods

This retrospective cohort study included two populations of veterans in VA care in the United States during 2004 and 2005: 1) HIV-infected veterans; and 2) age, race and facility-matched uninfected comparators.

We used data from the Veterans Aging Cohort Study (VACS), a prospective, observational cohort study of HIV infected veterans and age, race and site matched controls. VACS is made up of eight urban sites, but also includes a virtual cohort (VACS-VC), a nationally representative sample of HIV-infected veterans and matched uninfected controls identified through the VA electronic medical record (EMR) system and followed since 1996 [17,18]. Demographic data, medical diagnoses (based on International Classification of Diseases, Ninth Revision, Clinical Modification [ICD-9-CM] codes), and laboratory results were extracted from VA EMR. Medicare and/or Medicaid enrollment and utilization for these veterans were extracted from data provided by the Center for Medicare & Medicaid Services (CMS). The study received approval from Human Investigations Committee at Yale University and the VA Connecticut Healthcare System and was granted a waiver of informed consent.

**Figure 1:**
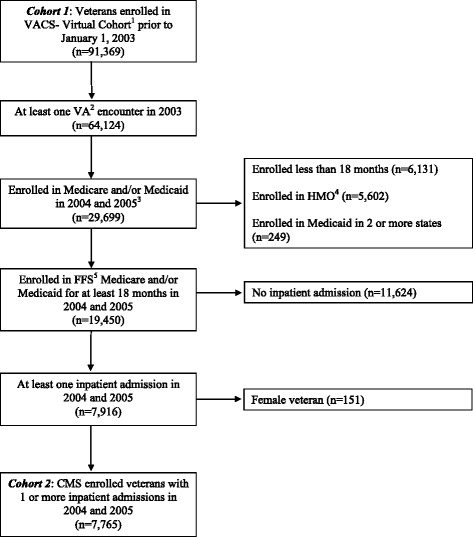
**Cohort development for 2004 dual enrollment and dual use in 2004-2005.** ^1^Veterans Aging Cohort Study Virtual Cohort. ^2^Veterans Administration. ^3^4827 veterans excluded because died in 2004 or 2005. ^4^Health Maintenance Organization. ^5^Fee-for-service.

We started with a cohort that included all veterans enrolled in the VACS-VC prior to January 1, 2003 (cohort 1 in Figure 1) and engaged in VA care. For the purpose of this analysis we defined engaged in care as at least one inpatient or outpatient visit to a VA medical center in 2003. Using CMS enrollment data we determined the frequency of Medicare and/or Medicaid enrollment in this cohort. To study frequency and drivers of CMS use we limited our cohort of VA enrolled veterans to include only those with coverage for CMS use. We narrowed our sample to veterans that were CMS enrolled in the 2004 and 2005 calendar years and had at least one inpatient admission in either VA or non-VA hospitals during this same period. From this group we excluded those CMS enrolled for less than 18 months in the 24 month period, enrolled in HMO plans (as defined in the CMS data file), and enrolled in Medicaid in more than one state. Finally, we excluded female veterans, due to their low numbers in our final sample (cohort 2 in Figure 1). Our final sample consisted of 7,765 male HIV infected and uninfected veterans enrolled in FFS Medicare and/or Medicaid with at least one inpatient admission in 2004 and 2005. We used this cohort to determine the frequency and predictors of non-VA hospital admissions.

We created a series of variables to identify factors associated with non-VA hospital admissions among male veterans, enrolled in fee for service (FFS) Medicare and/or Medicaid that received inpatient care in 2004 and 2005 (cohort 2). Our dependent variable was a dichotomous indicator of any non-VA admissions, (vs. only VA inpatient admissions). Hospital admissions were identified by combining VA EMR data with CMS FFS reimbursement files.

We considered a number of independent variables we believed could influence use of non-VA inpatient care, including demographics, access to care, illness severity, and admission to a medical or surgical intensive care unit (ICU). Age and race data were taken from VA administrative databases. Age was constructed as a dichotomous variable, under age 65 and 65 years and older. Race was divided into three categorical variables: White, African American, and Other.

Patients’ access to care was measured by three variables: enrollment in Medicare, Medicaid or both programs; distance to nearest VA tertiary care center treated as a categorical variable: 0 to 9.9 miles, 10-29.9 miles, 30-59.9 miles, 60-119.9 miles, and greater than 120 miles [19]; and an indicator of whether veterans were subject to co-pays for VA care. Co-pay status was in turn determined using data on service connection, medical conditions, and an income-based means test for each veteran.

We used the CMS hierarchical condition categories (HCC) model to calculate risk scores [20] and used the scores as a measure of patients’ illness severity. The HCC model is a risk adjustment method employed by CMS to predict prospective payments for patients based on the previous year’s claims data, using patient demographics and major medical conditions, and has been applied to the VA population in the past for similar analyses [7]. Each year the average risk score is set at one, and we divided our HCC risk scores into a dichotomous variable: scores greater than one and scores less than or equal to one. We created a binary variable for HIV status, identified through VA administrative data. We also used VA administrative data to identify veterans that carried a diagnosis of a substance use problem (illicit substance abuse or dependence) using ICD-9CM codes and a previously validated algorithm available on the VACS website (vacohort.org).

We generated categorical variables for number of inpatient admissions (one vs. two or more) and the admission diagnosis (ICU vs. non-ICU admission) because we believe both could influence the likelihood of a veteran having a non-VA admission. Veterans with more frequent admissions have more “opportunities” to be hospitalized in a non-VA facility. Similarly, patients in critical condition, requiring admission to ICUs, may be more likely to be routed to a non-VA hospital. Though these variables do not reflect patient behavior, they may influence the likelihood of an admission outside the VA and, thus, we controlled for these factors in our model.

### Analysis

We first examined veterans in the VACS-VC (cohort 1) to determine the frequency of CMS enrollment among veterans engaged in care. We stratified our analysis by age and HIV status, and used student t-tests to compare the frequency of CMS enrollment between HIV-infected and uninfected veterans. Next, we described the cohort of male, veterans enrolled in FFS Medicare and/or Medicaid with at least one inpatient admission (cohort 2) using the independent variables listed above. We stratified cohort 2 by HIV status and used chi-squared tests to compare the two sub-populations. Finally, we explored the association between these independent variables and non-VA hospital admissions using univariate and multivariable logistic regression. We created a multivariate model that included all patients in cohort 2, as well as separate models stratified by HIV status. All analyses were conducted using SAS version 9.2.

## Results

Overall, HIV-infected veterans engaged in VA care were more likely than uninfected veterans to be enrolled in Medicare and/or Medicaid (38% vs. 33%, p < 0.01) (Table 1). While HIV-infected veterans under age 65 were more likely to be CMS enrolled than uninfected veterans, (36% vs. 29%, p < 0.01), HIV-infected veterans 65 years and older were less likely to be CMS enrolled (53% vs. 70%, p < 0.01). The majority of CMS enrolled veterans were enrolled in Medicare, but HIV-infected veterans were more likely than uninfected veterans to be enrolled in Medicaid, either alone or in combination with Medicare.

**Table 1:** **Medicare and/or Medicaid enrollment among veterans enrolled in VACS-VC** ^**1**^ **in 2004**

	**All patients** ***n (%)***	**HIV+** ***n (%)***	**HIV-** ***n (%)***	**p-value**
	**n = 91369**	**n = 29909**	**n = 61460**	
**Total Unique in VACS-VC**				
**Medicare Only**	20964 (23)	6461 (22)	14503 (24)	<.0001
**Medicaid Only**	5220 (6)	2203 (7)	3017 (5)	<.0001
**Medicare and Medicaid**	5290 (6)	2618 (9)	2672 (4)	<.0001
**Total Enrolled in CMS** ^**2**^	31474 (34)	11282 (38)	20192 (33)	<.0001
**Under 65**	n = 82835	n = 27193	n = 55642	
**Medicare Only**	16309 (20)	5334 (20)	10975 (20)	0.7111
**Medicaid Only**	5187 (6)	2190 (8)	2997 (5)	<.0001
**Medicare and Medicaid**	4475 (5)	2329 (9)	2146 (4)	<.0001
**Total Enrolled in CMS**	25971 (31)	9853 (36)	16118 (29)	<.0001
**65 and over**	n = 8534	n = 2716	n = 5818	
**Medicare Only**	4655 (55)	1127 (41)	3528 (61)	<.0001
**Medicaid Only**	33 (0)	13 (0)	20 (0)	0.3497
**Medicare and Medicaid**	815 (10)	289 (11)	526 (9)	0.0192
**Total Enrolled in CMS**	5503 (64)	1429 (53)	4074 (70)	<.0001

^1^Veterans Aging Cohort Study – Virtual Cohort.

^2^Center for Medicare & Medicaid Service.

Among veterans enrolled in FFS Medicare and/or Medicaid with at least one inpatient admission (cohort 2) the frequency of HIV-infected veterans with CMS use was lower than the frequency among uninfected veterans (48% vs.54%, p < 0.01) (Table 2). Compared to uninfected veterans, HIV-infected veterans were younger and lived closer to VA hospitals. A greater proportion of HIV-infected veterans was also of African American race, had an HCC score greater than 1 and carried a diagnosis of substance use problem.

**Table 2:** **CMS** ^**1**^ **enrolled veterans with 1 or more inpatient admissions in 2004 and 2005**

	**All patients** ***n (%)***	**HIV+** ***n (%)***	**HIV-** ***n (%)***	**p-value**
	**n = 7765**	**n = 2799**	**n = 4966**	
**DEMOGRAPHICS**				
**Age**				
**<65**	6319 (81)	2405 (86)	3914 (79)	<.0001
**≥65**	1446 (19)	394 (14)	1052 (21)	
**Race**				
**White**	3903 (50)	1343 (48)	2560 (52)	0.009
**African American**	3755 (48)	1413 (50)	2342 (47)	
**Other**	107 (2)	43 (2)	64 (1)	
**Distance from VA**				
**0-9.9 miles**	3253 (42)	1445 (52)	1808 (36)	<.0001
**10-29.9 miles**	1824 (24)	599 (21)	1225 (25)	
**30-59.9 miles**	1270 (16)	393 (14)	877 (18)	
**60-119.9 miles**	1192 (15)	310 (11)	882 (18)	
**≥120 miles**	224 (3)	52 (2)	172 (3)	
**Co-pay status**				
**No Co-pay**	7174 (95)	2592 (96)	4582 (95)	0.0997
**Co-pay**	352 (5)	112 (4)	240 (5)	
**ENROLLMENT**				
**Medicare Only**	6408 (83)	2088 (74)	4320 (87)	<.0001
**Medicaid Only**	469 (6)	274 (10)	195 (4)	
**Medicare and Medicaid**	888 (11)	437 (16)	451 (9)	
**HEALTH**				
**HIV**				
**Positive**	2799 (36)			
**Negative**	4966 (64)			
**HCC Risk Score** ^**2**^				
**<1**	4129 (53)	1040 (37)	3089 (62)	<.0001
**≥1**	3636 (47)	1759 (63)	1877 (38)	
**INPATIENT ADMISSIONS**				
**Number of Admissions**				
**1**	3186 (41)	1091 (39)	2095 (42)	0.0058
**≥2**	4579 (59)	1708 (61)	2871 (58)	
**ICU** ^**3**^ **Admission**				
**Yes**	1637 (21)	575 (21)	1062 (21)	0.3822
**No**	6128 (79)	2224 (79)	3904 (79)	
**Substance Abuse**				
**Yes**	1958 (25)	828 (30)	1130 (23)	<.0001
**No**	5807 (75)	1971 (70)	3836 (77)	
**UTILIZATION**				
**VA** ^**4**^ **Admissions Only**	3757 (48)	1467 (52)	2290 (46)	<.0001
**Non-VA Admission**	4008 (52)	1332 (48)	2676 (54)	

^1^Center for Medicare & Medicaid Serivces.

^2^Hierarchical Conditional Category Risk Score.

^3^Intensive Care Unit.

^4^Veterans Administration.

HIV-infected veterans had lower odds of CMS use than uninfected veterans (OR = 0.78 [95% CI: 0.71, 0.85]) (Table 3). In addition, older veterans and veterans with higher HCC risk scores had greater odds of CMS use than their respective younger and healthier counterparts. Among HIV-infected veterans a substance abuse diagnosis was associated with increased non-VA use (OR = 1.34 [95% CI: 1.14, 1.58]). There was not a significant association between substance abuse and non-VA use in uninfected veterans (OR = 0.91 [95% CI: 0.80, 1.04]).

**Table 3:** **Characteristics associated with non-VA inpatient admissions in 2004 and 2005, univariate analysis**

	**All patients** ***n (%)***	**p-value**	**HIV+** ***n (%)***	**p-value**	**HIV-** ***n (%)***	**p-value**
**DEMOGRAPHICS**						
**Age**						
**<65**	Ref		Ref		Ref	
**≥65**	1.86 (1.65-2.09)	<.0001	1.39 (1.12-1.72)	0.0028	2.05 (1.77-2.36)	<.0001
**Race**						
**White**	Ref		Ref		Ref	
**African American**	0.96 (0.87-1.04)	0.3126	1.08 (0.93-1.26)	0.3031	0.90 (0.81-1.01)	0.0696
**Other**	3.40 (2.13-5.41)	<.0001	3.39 (1.70-6.79)	0.0006	3.58 (1.90-6.74)	<.0001
**Distance from VA**						
**0-9.9 miles**	Ref		Ref		Ref	
**10-29.9 miles**	1.30 (1.16-1.46)	<.0001	1.16 (0.96-1.40)	0.1297	1.37 (1.19-1.59)	<.0001
**30-59.9 miles**	1.75 (1.54-2.00)	<.0001	1.45 (1.16-1.81)	0.0012	1.91 (1.62-2.25)	<.0001
**60-119.9 miles**	2.55 (2.22-2.93)	<.0001	1.92 (1.49-2.46)	<.0001	2.82 (2.38-3.34)	<.0001
**≥120 miles**	2.41 (1.82-3.20)	<.0001	2.95 (1.62-5.37)	0.0004	2.26 (1.63-3.12)	<.0001
**Co-pay status**						
**No Co-pay**	Ref		Ref		Ref	
**Co-pay**	2.58 (2.04-3.27)	<.0001	1.92 (1.30-2.84)	0.001	3.01 (2.22-4.08)	<.0001
**ENROLLMENT**						
**Medicare**	Ref		Ref		Ref	
**Medicaid**	1.94 (1.60-2.36)	<.0001	2.51 (1.94-3.27)	<.0001	1.75 (1.30-2.36)	0.0003
**Medicare and Medicaid**	2.40 (2.07-2.79)	<.0001	2.95 (2.38-3.68)	<.0001	2.26 (1.83-2.80)	<.0001
**HEALTH**						
**HIV**						
**Positive**	0.78 (0.71-0.85)	<.0001				
**Negative**	Ref					
**HCC Risk Score** ^**1**^						
**<1**	Ref		Ref		Ref	
**≥1**	1.14 (1.04-1.25)	0.004	1.26 (1.08-1.46)	0.0039	1.21 (1.08-1.36)	0.0014
**INPATIENT ADMISSIONS**						
**Number of Admissions**						
**1**	Ref		Ref		Ref	
**≥2**	3.06 (2.79-3.37)	<.0001	4.29 (3.64-5.06)	<.0001	2.64 (2.35-2.97)	<.0001
**ICU** ^**2**^ **Admission**						
**Yes**	2.21 (1.97-2.47)	<.0001	2.00 (1.66-2.42)	<.0001	2.34 (2.02-2.70)	<.0001
**No**	Ref		Ref		Ref	
**Substance Abuse**						
**Yes**	1.04 (0.94-1.15)	0.4531	1.34 (1.14-1.58)	0.0004	0.91 (0.80-1.04)	0.1549
**No**	Ref		Ref		Ref	

^1^Hierarchical Conditional Category Risk Score.

^2^Intensive Care Unit.

The association between HIV status and decreased odds of non-VA use remained in the multivariable model (OR = 0.76 [95% CI: 0.68, 0.85]); however, there was no longer an association between higher HCC risk score and increased non-VA use (Table 4). We posited that number of admissions, which had the greatest marginal effect on the multivariate model, was overwhelming the effects of other variables. When we removed number of admissions from the adjusted model (analysis not shown), both higher HCC risk score and African American race had significant associations with increased non-VA use (OR = 1.28 [95% CI: 1.13, 1.44] and OR = 1.18 [95% CI: 1.05, 1.33], respectively).

**Table 4:** **Characteristics associated with non-VA inpatient admissions in 2004 and 2005, multivariate analysis**

	**All patients** ***n (%)***	**p-value**	**HIV+** ***n (%)***	**p-value**	**HIV-** ***n (%)***	**p-value**
**DEMOGRAPHICS**						
**Age**						
**<65**	Ref		Ref		Ref	
**≥65**	1.90 (1.65-2.17)	<.0001	1.61 (1.25-2.08)	0.0002	2.00 (1.70-2.35)	<.0001
**Race**						
**White**	Ref		Ref		Ref	
**African American**	1.05 (0.94-1.16)	0.4032	1.02 (0.86-1.22)	0.8293	1.07 (0.94-1.22)	0.3128
**Other**	3.17 (1.91-5.27)	<.0001	3.58 (1.63-7.85)	0.0015	2.98 (1.52-5.83)	0.0015
**Distance from VA**						
**0-9.9 miles**	Ref		Ref		Ref	
**10-29.9 miles**	1.51 (1.33-1.72)	<.0001	1.41 (1.13-1.75)	0.0022	1.59 (1.35-1.86)	<.0001
**30-59.9 miles**	2.08 (1.79-2.41)	<.0001	1.78 (1.38-2.30)	<.0001	2.25 (1.87-2.70)	<.0001
**60-119.9 miles**	3.42 (2.92-4.01)	<.0001	3.06 (2.29-4.09)	<.0001	3.62 (2.99-4.37)	<.0001
**≥120 miles**	3.06 (2.23-4.19)	<.0001	5.67 (2.90-11.06)	<.0001	2.61 (1.83-3.74)	<.0001
**Co-pay status**						
**No Co-pay**	Ref		Ref		Ref	
**Co-pay**	2.66 (2.05-3.46)	<.0001	2.61 (1.68-4.05)	<.0001	2.76 (1.99-3.84)	<.0001
**ENROLLMENT**						
**Medicare**	Ref		Ref		Ref	
**Medicaid**	2.83 (2.28-3.52)	<.0001	2.93 (2.17-3.94)	<.0001	2.57 (1.85-3.57)	<.0001
**Medicare and Medicaid**	2.50 (2.11-2.95)	<.0001	2.89 (2.27-3.69)	<.0001	2.17 (1.72-2.73)	<.0001
**HEALTH**						
**HIV**						
**Positive**	0.76 (0.68-0.85)	<.0001				
**Negative**	Ref					
**HCC Risk Score** ^**1**^						
**<1**	Ref		Ref		Ref	
**≥1**	0.99 (0.89-1.10)	0.8071	0.95 (0.79-1.14)	0.5937	1.01 (0.88-1.15)	0.9127
**INPATIENT ADMISSIONS**						
**Number of Admissions**						
**1**	Ref		Ref		Ref	
**≥2**	3.10 (2.79-3.45)	<.0001	4.45 (3.69-5.37)	<.0001	2.62 (2.30-2.98)	<.0001
**ICU** ^**2**^ **Admission**						
**Yes**	1.73 (1.52-1.96)	<.0001	1.42 (1.15-1.76)	0.0012	1.94 (1.65-2.27)	<.0001
**No**	Ref		Ref		Ref	
**Substance Abuse**						
**Yes**	1.16 (1.03-1.31)	0.0179	1.28 (1.05-1.55)	0.0138	1.08 (0.92-1.26)	0.3356
**No**	Ref		Ref		Ref	

^1^Hierarchical Conditional Category Risk Score.

^2^Intensive Care Unit.

After stratifying by HIV statuses, the predictors of CMS use were similar in both the HIV-infected and uninfected models (Table 4).

## Discussion

In our sample of veterans engaged in VA care, we found that HIV-infected veterans were more likely than their uninfected counterparts to be enrolled in Medicare and/or Medicaid, but this difference varied by age. HIV-infected veterans under age 65 were more likely to be CMS enrolled, while HIV-infected veterans 65 years and older were much less likely to be CMS enrolled. Confirming our initial hypothesis, we also found that male, HIV-infected veterans enrolled in FFS Medicare and/or Medicaid were less likely to have an outside hospitalization. This was despite the fact that these veterans were generally sicker, more likely to have multiple admissions, and more likely to be enrolled in FFS Medicaid – independent risk factors for greater non-VA use.

We suspect that HIV-infected veterans were less likely to use non-VA hospitals because they feel more tied to the VA – possibly due to the integrated care and the different providers they see in VA infectious disease clinics or the desire for continuity with established caregivers for what is historically a stigmatized disease. The prescription drug coverage afforded veterans – and the high costs of antiretroviral therapy - may also play a significant role. While every state provides some coverage for antiretroviral drugs, the coverage varies substantially. Very few states cover as many of these drugs as are covered nationally within VA. This conclusion is also supported from patient self report data in VACS 8 in which 96% of those on ART reported getting all their antiretroviral medications through VA [17].

Our findings on frequency of CMS enrollment among veterans were not completely consistent with previous literature. Overall, we found lower rates of Medicare enrollment, but higher rates of enrollment in Medicaid [21]. This difference was particularly evident among veterans 65 years and older where Medicare enrollment has been reported to be greater than 90% [7,21]. This difference is likely driven primarily by our initial cohort (cohort 1), which included only patients who were already engaged in VA care, i.e. were seen at the VA at least once in the past year. We suspect that veterans already receiving care at the VA are less likely to seek alternative forms of coverage than all veterans ever enrolled in VA care. The difference may also be partially attributed to the greater share of HIV-infected veterans in our sample than in previously studied groups. VACS-VC is a nationally representative sample of HIV-infected veterans, not all veterans enrolled in VA care. As noted in the background, HIV-infected veterans differentially qualify for Medicare coverage, primarily through disability [10]. Similarly, we believe that comorbid and socioeconomic factors that contribute to higher rates of Medicaid enrollment among all persons living with HIV contributed to the higher rates of Medicaid enrollment in our sample of veterans. Among HIV infected and uninfected veterans, some of these changes may also be partially driven by a change in the population utilizing VA care since 1999 [8,21], when Shen, et al. reported their data, and our results from five years later, and may also reflect an evolving perception of VA care or increased Medicare cost sharing.

Our findings regarding predictors of non-VA hospitalization among CMS enrolled veterans engaged in VA care were consistent with previous literature. In general, these studies have found older age, increased distance to the VA, greater cost-sharing and worse health to be associated with non-VA use [7,8,22,23]. Our results confirm these findings among HIV-infected veterans, and provide guidance to VA care providers on patients that are more likely to receive non-VA care and thus benefit from care coordination efforts with outside providers.

Our results should be viewed in light of several limitations – most importantly, our limited access to data on private insurance enrollment and utilization in the VA population. As a result of the lack of claims data for patients hospitalized under private insurance, some patients classified as having only VA admissions may actually have unaccounted for admissions to non-VA hospitals. Moreover, CMS enrollment with private insurance may lead to significantly lower costs for veterans, influencing their choice of health care setting. The VACS does collect some survey data on private insurance enrollment. Among veterans in VACS8, 15% of HIV-infected veterans and 20% of uninfected veterans reported enrollment in private insurance (analysis not shown). Given the lower rates of supplemental insurance in the HIV-infected population, we believe our finding that HIV-infected veterans are less likely to have non-VA admissions likely still holds true. Another limitation is that inpatient admissions are more likely to be influenced by factors beyond patient preference. We tried to control for those variables in our analysis, but using inpatient admissions as our outcome may limit the conclusions that can be drawn from our results. We chose inpatient admissions because non-VA inpatient care places HIV-infected veterans at risk for adverse events related to poor coordination of care transitions between outside hospitals and VHA HIV clinics; however, choice in outpatient setting may be a better reflection of patient preferences and will be the subject of future studies. We also did not take into account pathways to CMS enrollment. Veterans that were enrolled in Medicare or Medicaid prior to engagement with the VA system may be more likely to have non-VA admissions; the opposite may be true of veterans that were seeking care with the VA prior to enrolling with Medicare and/or Medicaid. In addition, though most HIV infected people with Medicare qualify through disability, it is unknown if this is also true among veterans.

The heterogeneity of Medicare and/or Medicaid from state to state further limits our ability to generalize the data. Medicare Advantage plans, i.e. managed care plans provided by private insurers, are highly variable by design, and Medicaid benefits and eligibility vary from state to state. Moreover, to date only 26 of the 50 states have chosen to accept the ACA provision to expand Medicaid, making comparisons between states more difficult in the post-ACA era. Finally, our findings are limited by the age of our data. We restricted our analysis to 2004 and 2005, the latest years we had complete data available. However, we believe our analysis uses more recent data than similar studies analyzing CMS enrollment and CMS use.

## Conclusion

Many veterans engaged in care in the VA are also enrolled in Medicare and/or Medicaid, including a significant proportion of veterans under age 65. Like the general population, the rates of CMS enrollment are higher among HIV infected veterans. However, among veterans enrolled in both VA and CMS care, HIV-infected veterans have lower odds of non-VA hospitalization despite having greater illness severity and more frequent hospitalizations than uninfected veterans. The VA’s integrated model of care used to treat HIV/AIDS may explain why HIV-infected veterans were more likely to seek care within the VA.

The impact of CMS use on veterans with HIV and other complex medical conditions requires further investigation. HIV-infected veterans have complicated medical histories and are likely to experience inefficient care and poor outcomes related to non-VA use. Research that provides evidence of the cost and health implications of CMS use may guide the VA in how aggressively to pursue and retain these patients in the VA system.

Future research should also explore why veterans with HIV were less likely than uninfected veterans to use non-VA care. If specific aspects of VA’s integrated care programs for veterans with HIV, such as intensive case management, are found to contribute to lower likelihood of non-VA admission, then this would inform strategies to limit non-VA use among other veteran populations at high risk from fragmented care, such as veterans with other major chronic diseases or illnesses.

CMS enrollment is a valuable resource for veterans who face higher copayments or live long distances from VA medical centers; however, among some veterans, CMS enrollment may lead to inefficient care and poor health outcomes. As health coverage is further expanded efforts should be made to ensure that additional coverage is reaching the populations that can benefit the most. The VA and federal government should shape their policies to incentivize use of a single health system and develop procedures to ensure comprehensive exchange of information between health systems.

## Abbreviations

HIV: Human immunodeficiency virus
AIDS: Acquired immunodeficiency disease syndrome
VA: Veterans Administration
CMS: Center for Medicare & Medicaid Services
ACA: Affordable Care Act
VACS-VC: Veterans Aging Cohort Study – Virtual Cohort
FFS: Fee for service
ICU: Intensive care unit
HCC: Hierarchical condition category
95% CI: 95% Confidence interval
OR: Odds ratio
